# DDID: a comprehensive resource for visualization and analysis of diet–drug interactions

**DOI:** 10.1093/bib/bbae212

**Published:** 2024-05-06

**Authors:** Yanfeng Hong, Hongquan Xu, Yuhong Liu, Sisi Zhu, Chao Tian, Gongxing Chen, Feng Zhu, Lin Tao

**Affiliations:** Key Laboratory of Elemene Class Anti-cancer Chinese Medicines, School of Pharmacy, Hangzhou Normal University, Hangzhou 311121, China; Key Laboratory of Elemene Class Anti-cancer Chinese Medicines, School of Pharmacy, Hangzhou Normal University, Hangzhou 311121, China; Key Laboratory of Elemene Class Anti-cancer Chinese Medicines, School of Pharmacy, Hangzhou Normal University, Hangzhou 311121, China; Key Laboratory of Elemene Class Anti-cancer Chinese Medicines, School of Pharmacy, Hangzhou Normal University, Hangzhou 311121, China; Key Laboratory of Elemene Class Anti-cancer Chinese Medicines, School of Pharmacy, Hangzhou Normal University, Hangzhou 311121, China; Key Laboratory of Elemene Class Anti-cancer Chinese Medicines, School of Pharmacy, Hangzhou Normal University, Hangzhou 311121, China; College of Pharmaceutical Sciences, The Second Affiliated Hospital, Zhejiang University School of Medicine, Zhejiang University, Hangzhou 310058, China; Innovation Institute for Affiliated Intelligence in Medicine of Zhejiang University, Alibaba-Zhejiang University Joint Research Center of Future Digital Healthcare, Hangzhou 330110, China; Key Laboratory of Elemene Class Anti-cancer Chinese Medicines, School of Pharmacy, Hangzhou Normal University, Hangzhou 311121, China

**Keywords:** database, daily intake, drug safety, diet–drug interactions, bioinformatics

## Abstract

Diet–drug interactions (DDIs) are pivotal in drug discovery and pharmacovigilance. DDIs can modify the systemic bioavailability/pharmacokinetics of drugs, posing a threat to public health and patient safety. Therefore, it is crucial to establish a platform to reveal the correlation between diets and drugs. Accordingly, we have established a publicly accessible online platform, known as Diet-Drug Interactions Database (DDID, https://bddg.hznu.edu.cn/ddid/), to systematically detail the correlation and corresponding mechanisms of DDIs. The platform comprises 1338 foods/herbs, encompassing flora and fauna, alongside 1516 widely used drugs and 23 950 interaction records. All interactions are meticulously scrutinized and segmented into five categories, thereby resulting in evaluations (positive, negative, no effect, harmful and possible). Besides, cross-linkages between foods/herbs, drugs and other databases are furnished. In conclusion, DDID is a useful resource for comprehending the correlation between foods, herbs and drugs and holds a promise to enhance drug utilization and research on drug combinations.

## INTRODUCTION

The effectiveness of medication is influenced by various interacting factors, including drug–drug interactions, drug–natural product interactions and drug–diet interactions (DDIs) [1–4]. Nevertheless, DDIs often can be neglected [5]. In addition to foods, herbs (including nutraceuticals, dietary natural products, *etc.*) are also commonly consumed today. As highlighted by the World Health Organization, approximately 70% of the world’s population currently uses herbal medicine as a complementary or alternative treatment [6]. The common use of foods and herbs in combination with medicines can potentially interact, influencing the medicines’ effectiveness [7, 8]. Hence, understanding the effects and mechanisms of various DDIs is crucial. In our research, foods mainly consist of items consumed by humans to sustain life, provide energy, promote growth (such as grains, vegetables, fruits), while herbs primarily pertain to dietary supplements like herbal products, nutritional supplements to benefit health [9]. Therefore, DDIs are classified into food–drug interactions (FDIs) and herb–drug interactions (HDIs) [7, 8].

For instance, foods (e.g. cranberry) or herbs (e.g. *Matricaria chamomilla*, *Hypericum perforatum*) can affect warfarin, leading to a reduced anticoagulant effect [10, 11]. Simvastatin’s effectiveness can be potentiated by grapefruit or diminished by *Ginkgo biloba* extract [12, 13]. Conversely, *G. biloba* extract enhances the therapeutic effect of cisplatin [14]. The United States Food and Drug Administration (FDA) has released experimental recommendations for potential FDIs. By utilizing the area under the curve or maximum plasma concentration (Cmax) in pharmacology, a bioequivalence range of 80–125% between the two is applied within the 90% confidence interval range of the total exposure. Any combination beyond this range may have a positive or negative therapeutic effect on the drug [15].

To date, several DDI databases or datasets have been established. The majority of them focus on processing data from Drugbank [16] (like DFinder [17], FDMine [18]), which provides 1195 records (Drugbank 5.0) for the information of DDIs; some describe the impact of each herb supplement on drug efficacy (such as Supp.AI [19], NaPDI [20]); and the other two provide datasets on food combinations affecting drug bioavailability (like Harriet Bennett-Lenane [21], Daniel Reker [22]). Special mention should be made of FooDrugs, the latest and largest FDI database, with over one million food-drug-related entries [23]. However, its data are largely generated by computational methods and lack sufficient manual review. Although these studies related to DDIs have unique research value, DDIs have not been comprehensively summarized by any existing databases (Table 1). Therefore, it is crucial to develop a reliable platform capable of querying DDIs.

**Table 1:** A variety of open-access databases (including datasets) are available for providing the data on DDIs (the first is the new database proposed in this study, while the remaining ones are sorted based on the category of interactions they contain)

**Database**	**Number of interactions**	**Specific food–drug interactions**	**Food combination–drug interactions**	**Herb–drug interactions**	**All manually curated**
DDID^a^	23 950	√	√	√	√
Drugbank 5.0^b^	1195	√	√	√	√
DFI corpus^c^	2498	√	×	×	×
NutriChem 2.0^d^	14 662^e^	√	×	√	×
DFinder^f^	15 890^e^	√	×	×	×
Sup.AI^g^	59 096^e^	√	×	√	×
FDMine^h^	87 192^e^	√	×	×	×
FooDrugs^i^	1108 429	√	×	×	×
Harriet Bennett-Lenane^j^	141	×	√	×	√
NaPDI^k^	140	×	×	√	√

The existence and non-existence of certain data types were indicated using ‘√’ and ‘×’, respectively.

^a^ https://bddg.hznu.edu.cn/ddid/

^b^ https://go.drugbank.com/

^c^ https://github.com/ccadd-snu/corpus-for-DFI-extraction

^d^ http://sbb.hku.hk/services/NutriChem-2.0/

^e^This dataset includes the food ingredients–drugs interactions, instead of single food or herbs.

^f^ https://github.com/23AIBox/23AIBox-DFinder

^g^ https://supp.ai/

^h^ https://github.com/mostafiz67/FDMine_Framework

^i^ https://zenodo.org/records/8192515

^j^ https://pubmed.ncbi.nlm.nih.gov/34563654/

^k^ https://napdicenter.org/recommended-approaches/

In this study, we developed a DDI database named ‘DDID’ to integrate information from various sources. Data related to DDIs are manually extracted and systematically reviewed from PubMed or the FDA Label; next, the interaction effects were evaluated according to FDA standards [15]; lastly, the information, including foods/herbs and drugs, is then retained on DDID while being fully referenced by linking to other publicly accessible databases [24–40]. In summary, DDID provides valuable resources for understanding the interaction mechanisms between foods/herbs and drugs, potentially advancing drug utilization and research on drug combinations [17, 18].

## CONSTRUCTION AND CONTENT

### Data source, collection and curation

**Figure 1:**
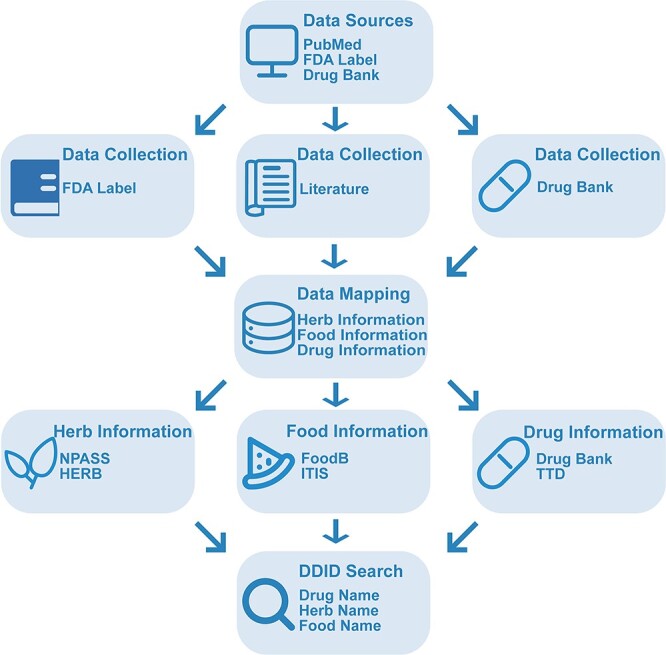
Workflow of DDID.

All data in DDID are retrieved from literature, FDA labels and various online repositories. The DDI information was obtained through the following sequential steps. Firstly, we employed keyword combinations to search for relevant literature from PubMed, including terms such as ‘drug + food + interactions’, ‘drug + herb + interactions’ and ‘drug + herbal ingredient + interactions’. Next, we selected, reviewed and extracted content of 1485 articles. Finally, the corresponding foods, herbs and drugs were mapped to the other popular databases. For FDA label, we reviewed the FDA Drugs webpage (http://www.accessdata.fda.gov), which contains information on approved oral drugs over the past 20 years. For DrugBank, we downloaded and parsed the SQL document, which contains sporadic information on DFIs [16] (Figure 1).

### Data standardization and platform implementation

**Figure 2:**
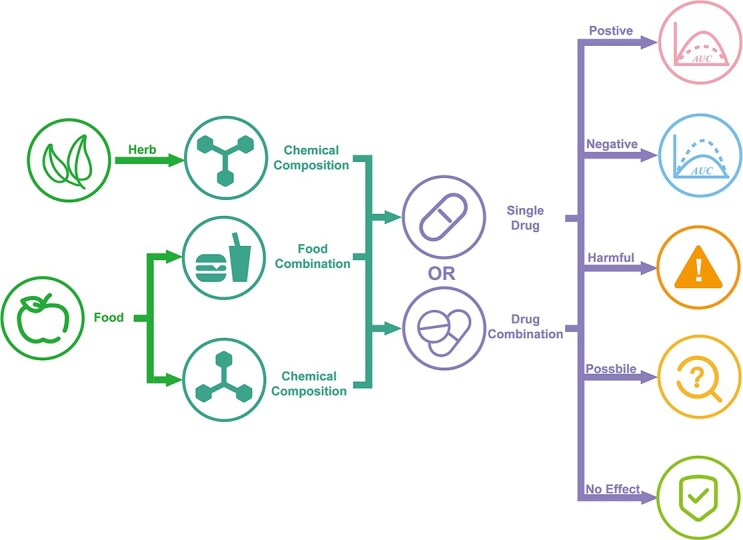
Flowchart of DDI relationships. The unique feature of DDID is to present the diet-drug interactions with reliable evaluations. The user can select three different aspects of the search (food, herb, or drug), according to their needs, all of which will provide comprehensive data and information.

To facilitate future readers’ access, application and analysis of DDID data, all originally collected information has been carefully cleaned and systematically standardized. The standardizations include the following: (a) most DDIs have information from different sources, mutually verified, and reliable; (b) all drugs, foods, herbs, compound components, species, potential targets and diseases indications linked with other popular databases (DrugBank [16], FoodB [24], Frida.fooddata.dk [41], IT IS [41], SymMap [29], NPASS [26], NCBI Taxonomy [27], Herb [25], PubChem [28], ChEBI [30], ADReCS [31], Uniport [42]). All DDIs are accessible for viewing, evaluation and download on the DDID website (Figure 2). Users can conduct free assessments without any login requirements.

### Data retrieval and access DDID

**Figure 3:**
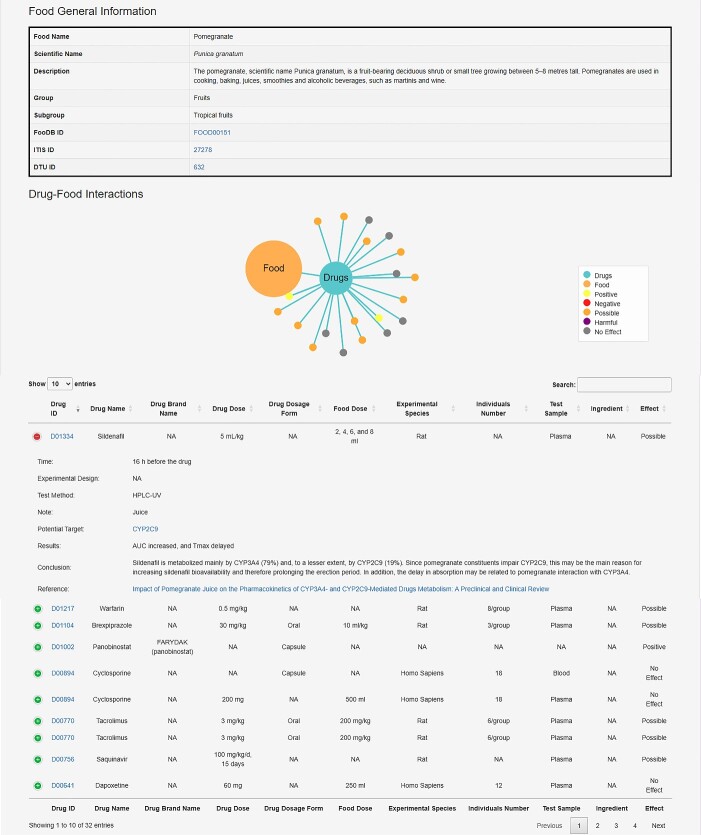
The food detail page of DDID provides general information on the food, interactions, and links to several other reliable databases.

The DDID employs a user-friendly online interface design and offers three distinct pages for searching and browsing information: drug, food and herb pages. The food page enables users to search for information on FDIs by typing in the food name or by selecting a food group. The first part of the response food detail page provides general information about relevant food species, including scientific names, species families and genera, as well as food classification and introduction. Additionally, external links to other databases are provided, including Taxonomy [27], FoodB [24], IT IS [41] and DTU [41]. The second part of the detail page lists the information on corresponding experiments, including experimental details, results and conclusion evaluations. This information can be toggled on and off by clicking on the row with a particular condition name. Users can click on the ‘Drug ID’ button to navigate to the medication page, which provides more detailed information about the drugs (Figure 3). Unlike the food page, both the drug and herbal pages contain unique basic information. For more details, please refer to the help page of the site (https://bddg.hznu.edu.cn/ddid/help/).

## CLARIFICATION AND DISCUSSION

### Category and analysis of interactions in DDID

**Figure 4:**
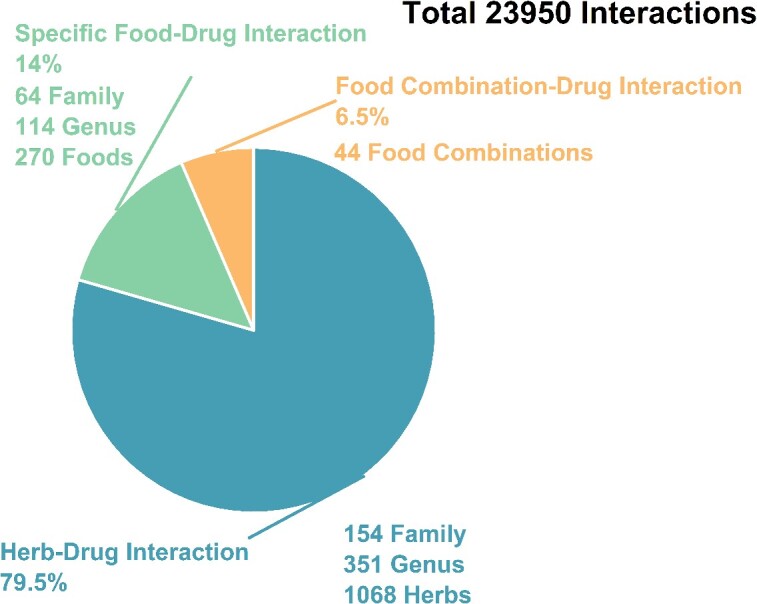
Pie charts illustrate the proportion of DDID's interactions accounted for by different sources of influence, describing the composition of the biological sources and their corresponding families, genera, and species numbers.

**Figure 5:**
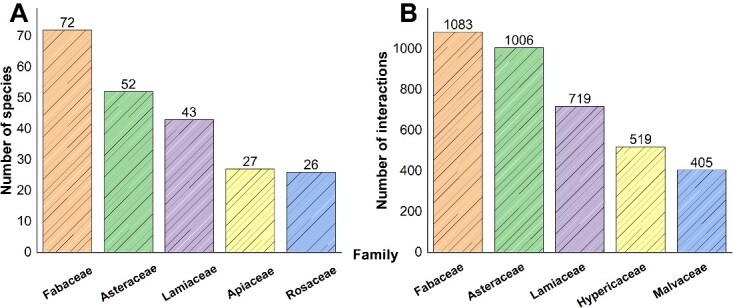
Statistical analysis of the top five plant families in DDID's herb sources. (A) the top five families and their species. (B) the top five families and their number of interactions.

In the context of known diet types, interactions can be divided into two categories: FDIs (including specific FDIs and food combination–drug interactions), and HDIs, totaling 23 915 interactions (Figure 4). For specific FDIs, DDID includes 212 unique foods and 14 common food ingredients (e.g. alcohol). It contains a large amount of interesting data, such as the possibility that turmeric in combination with tacrolimus may lead to increased edema and elevated creatinine levels [43]. Regarding food combination–drug interactions, there are literature reports that since 1 January 2010, approximately 40% (67 out of 157 identified products) of the drugs approved by the EMA and FDA have exhibited significant food effects or require the drug to be taken with or without food [44]. DDID provides 44 different meal modes. About HDIs, DDID currently includes 1068 herbs from 155 different families. Fabaceae represents the primary source of HDIs and encompasses numerous medicinal plants, including *Radix Puraria*, *Flos Sophorae*, *Radix Astragali* and *Radix Sophorae flavescentis*, which have received high attention in the pharmaceutical and healthcare sectors (Figure 5) [45].

### Explicitly classification of interaction effects

Despite being inconsistent in external conditions, DDID has assessed the impact of 23 915 interactions, classifying them as positive, negative, no effect, harmful and possible. In clinical trials, FDIs/HDIs can be defined as having positive or negative effects by assessing the bioequivalence [46]. Due to the low conversion rate of animal experiments (8%), evaluations are generally classified as having no effect and possible [47]. Harmful assessment is typically applied to interactions that suggest entail significant side effects, such as the intake of celery extract causing an elevation in serum venlafaxine levels in depression patients, leading to manic episodes [48].

### Interactions of foods/herbs ingredients and their targets

**Figure 6:**
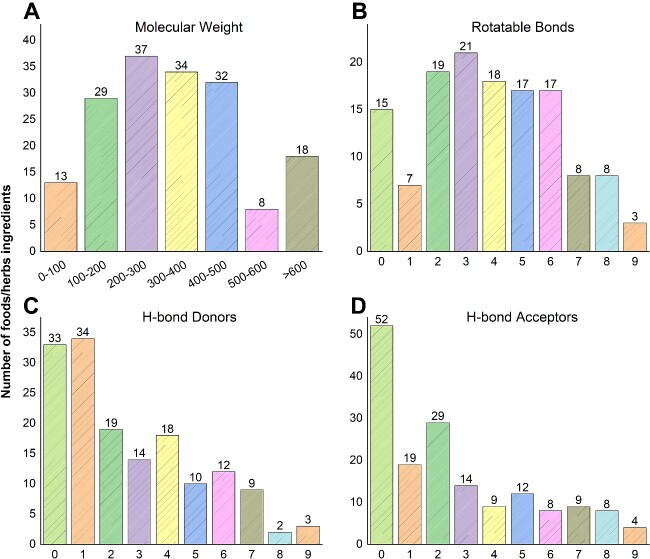
Distribution of the physio-chemical properties of compounds in DDID. (A) Molecular weight, (B) Rotatable Bonds, (C) H-bond donors, and (D) H-bond acceptors.

DDID currently contains 171 unique ingredients collected from foods and herbs, obtained from 321 scientific literature sources. All compounds were classified into their respective chemical categories using ClassyFire [49]. The top four superclasses are ‘Phenylpropanoids and polyketides’ (39.2%, 67), ‘Lipids and lipid-like molecules’ (20.5%, 35), ‘Organoheterocyclic compounds’ (9.35%, 16) and ‘Benzenoids’ (7.01%, 12) (Figure 6). A total of 112 different targets are involved in DDIs. For example, phenylpropanoids and polyketides; this superclass of compound involves 47.5% (11 207) of the DDIs. The primary targets of these interactions include Cytochrome P450 proteins (CYP) (54.7%, 10,947), P-glycoprotein (P-gp) (20.4%, 4087) and organic anion transporting polypeptides (OATP) (9.3%, 1869).

## APPLICATIONS OF THE DDID DATA

### Applications related to FDIs

Grapefruit juice can alter oral drug pharmacokinetics by different mechanisms, like immunosuppressants, antihistamines and central nervous system drugs. Upon searching for grapefruit in DDID, 228 drugs were identified. Interactions between grapefruit and drug can be divided into two categories, which are accomplished through the regulation of metabolic enzymes or transport proteins. Flavonoids and furanocoumarins present in grapefruit ingredients play a crucial role in these mechanisms [50]. Grapefruit juice can enhance the bioavailability and blood concentration of CYP3A4 metabolic related drugs by inhibiting CYP3A4 [51]. Additionally, grapefruit juice can reduce the bioavailability of aliskiren by inhibiting OATP1A2 [52], or modulate the effectiveness of drugs related to P-gp metabolism by inhibiting P-gp [51]. In addition, there is a common mechanism of FDIs known as physicochemical interaction. For instance, multivalent ions (such as calcium) present in milk can chelate with certain types of drugs (such as bisphosphonates and tetracyclines), hindering their absorption by the human body [53].

### Applications related to HDIs

The concurrent use of herbs may mimic, magnify or oppose the effect of drugs [7, 54]. With respect to HDIs, ginseng serves as an illustrative example. It can be used as an adjuvant to prescription drugs or as a daily health product, with various pharmacological activities [55]. Ginseng showed good effects in improving anxiety and depression scores and enhancing anti-fatigue ability when used as an adjuvant drug in combination with some anticancer drugs [56]. The combination of warfarin and ginseng may lead to a decrease in anticoagulant levels [57]. In rat experiments, combining ginseng with metformin may increase blood concentration or hypoglycemic ability, providing clinical reference value [58, 59].

### Potential of DDID in DDI prediction

Recent research in DDIs has highlighted the growing interest in applying artificial intelligence (AI) methods [17, 18, 21–23]. AI research in DDIs primarily focuses on text mining and prediction. However, existing data sources like DrugBank and FooDrugs face challenges of limited data and accuracy issues [16, 17]. Establishing a robust DDI database is crucial. In response, DDID provides a dataset of 23 950 interactions involving 1338 common foods/herbs and 1516 drugs, serving as a valuable resource for researchers. DDID aims to meet the demands of current HDIs and FDIs research.

## CONCLUSIONS

Understanding DDIs is invaluable, given their significant influence on drug efficacy through alterations in drug bioavailability, blood drug concentrations and other factors [17, 18]. Consequently, data provided by DDID (such as changes in clinical/experimental validation of bioavailability, side effects induced by combination in individual cases, changes in therapeutic efficacy, *etc.*) may promote drug utilization and research on drug combination. Moreover, we are committed to integrating high-quality data that emerge in the future into the DDID database.

**Key Points**

- Diet–drug interactions include food–drug interactions and herb–drug interactions.
- We developed a comprehensive database for these diet–drug interactions.
- The database comprised 23 950 interactions from 3013 literature reports.
- We analyzed ingredients related to diet–drug interactions, including chemical structures and their targets.
- Multiple structure- and text-based search functions were implemented.

## Data Availability

All DDID data can be freely downloaded at https://bddg.hznu.edu.cn/ddid/download.

## Author Biographies

**Yanfeng Hong, Hongquan Xu, Yuhong Liu, Sisi Zhu and Chao Tian** are Master students at School of Pharmacy, Hangzhou Normal University. They are specialized in diet–drug interactions and bioinformatics.

**Feng Zhu** is a tenured professor at the College of Pharmaceutical Sciences in Zhejiang University, China. His research group (https://idrblab.org/) has been working in the fields of NP-based drug discovery and bioinformatics.

**Lin Tao and Gongxing Chen** are professors at the School of Pharmacy in Hangzhou Normal University, China. They specialize in the areas of natural products, bioinformatics and machine learning.
